# Targeting the Gut Vascular Axis in Atherosclerosis: Probiotic and Phytoantioxidant Crosstalk as a Hypothesis-Generating Framework for Residual Cardiovascular Risk

**DOI:** 10.3390/ijms27198535

**Published:** 2026-09-24

**Authors:** Yen Chu, Kuo-Hsiung Huang, Chi-Nan Tseng

**Affiliations:** 1 Division of Thoracic and Cardiovascular Surgery, Chang Gung Memorial Hospital Linkou Branch, Taoyuan 33305, Taiwan; 2 Laboratory of Cardiovascular Physiology, Chang Gung Memorial Hospital Linkou Branch, Taoyuan 33305, Taiwan; 3 Department of Research and Development, Chang Gung Memorial Hospital Linkou Branch, No. 5, Fuxing St., Guishan Dist., Taoyuan 33305, Taiwan; 4 Department of Nursing, College of Nursing, Chang Gung University of Science and Technology, No. 261, Wenhua 1st Rd., Guishan Dist., Taoyuan 33303, Taiwan; 5 Graduate Institute of Traditional Chinese Medicine, The Medical College, Chang Gung University, No. 259, Wenhua 1st Rd., Guishan Dist., Taoyuan 33302, Taiwan; 6 Department of Laboratory Medicine, Section of Clinical Serology and Immunology, Chang Gung Memorial Hospital Linkou Branch, Taoyuan 33305, Taiwan

**Keywords:** atherosclerosis, gut–vascular axis, probiotics, phytoantioxidants, polyphenols, trimethylamine-N-oxide, short-chain fatty acids, endothelial dysfunction, Nrf2, SIRT1, sex differences, probiotic-strain specificity

## Abstract

Despite decades of effective statin therapy, a clinically important proportion of patients with atherosclerosis continue to experience ischemic events, even when low-density-lipoprotein cholesterol (LDL-C) is at guideline-recommended levels. This shortfall is increasingly attributed to residual inflammatory, oxidative, and gut microbial pathways that lipid lowering therapy was never designed to address. This review establishes the mechanistic rationale for combining two emerging dietary intervention classes, probiotics and phytoantioxidants, as adjuncts acting on the gut vascular axis. We systematically trace the evidence for each class. For probiotics, we elucidate the restoration of gut-barrier integrity, short-chain fatty acid (SCFA) signaling, bile acid metabolism, and suppression of the trimethylamine to trimethylamine-N-oxide (TMA-TMAO) axis, including strain-specific efficacy and sex-based disparities in microbial composition. For phytoantioxidants, we detail the activation of nuclear factor erythroid 2-related factor 2 (Nrf2), suppression of mitogen activated protein kinase (MAPK) and nuclear factor kappa B (NF-κB) signaling, and sirtuin 1 (SIRT1)-dependent endothelial protection. We critically appraise the biological plausibility of their combined administration while explicitly defining where this rationale remains a testable hypothesis rather than an established strategy. To date, only one small randomized trial has evaluated a combined probiotic and phytoantioxidant formulation against an atherosclerosis relevant biomarker, and it lacked single-agent comparator arms necessary to attribute benefit to synergy rather than individual components. Furthermore, evidence for each class alone remains constrained by small sample sizes and heterogeneous dosing. Finally, we propose a novel, sex-stratified, strain-specified combination trial framework designed to rigorously evaluate this dual-target approach. This synthesis is intended to generate testable hypotheses for future research rather than to establish an evidence base for current adjunctive clinical use.

## 1. Introduction

### 1.1. Atherosclerosis as a Chronic Inflammatory Vascular Disease

Atherosclerosis is now understood as a lipid-driven, immune-inflammatory vascular disease rather than a passive cholesterol deposition process. The disease begins with endothelial injury and is sustained by a self-perpetuating inflammatory program, which involves the recruitment of circulating leukocytes, subendothelial retention of apolipoprotein B-containing lipoproteins, and persistent local immune activation [1,2]. This distinction matters clinically as much as conceptually, because it is precisely the inflammatory component, not the lipid component, that current lipid-lowering therapy leaves largely untouched, and it is this gap that motivates the present review.

The cerebrovascular consequences of this process are considerable. Large-artery intracranial atherosclerotic disease is a major cause of ischemic stroke and stroke recurrence. In a recent retrospective, multicenter cohort of patients with symptomatic middle cerebral artery atherosclerotic disease, greater CT perfusion-defined hypoperfusion was associated with higher short-term recurrence risk, supporting further evaluation of hemodynamic impairment as a prognostic marker [3]. Anatomical risk stratification by stenosis grade alone, in other words, understates the true mechanistic burden.

### 1.2. The Gut–Vascular Axis: A Systemic Regulator of Vascular Health

A genuinely transformative development in cardiovascular medicine over the past decade has been the recognition of the gut–vascular axis as a systemic regulator of vascular homeostasis in its own right. The intestinal microbiota behaves, in effect, as a metabolically active organ: it generates bioactive metabolites that enter the systemic circulation and act directly on vascular tissue [4]. When this ecosystem is disturbed, a state generally termed dysbiosis and characterized by reduced microbial diversity and overgrowth of pathobiont taxa, the host metabolic profile may shift toward pro-atherogenic pathways, and observational data link such profiles to vascular disease [4].

A further critical consideration reframes our approach to current therapeutic strategies, as the gut microbiota interacts with lipid-lowering interventions through two distinct and separate mechanisms that must not be conflated. Beyond the influence of pharmacological agents, resident gut bacteria can convert a fraction of luminal cholesterol into the poorly absorbed sterol coprostanol via microbial cholesterol dehydrogenases, thereby providing a host-independent route of cholesterol elimination [5,6]. Emerging preclinical and early clinical data suggest that statin therapy can alter gut microbial composition and metabolite profiles through pathways distinct from hepatic 3-hydroxy-3-methylglutaryl-coenzyme A (HMG-CoA) reductase inhibition, and these changes may contribute to interindividual variability in statin response [7]. However, the extent to which these effects are microbiota-dependent in humans remains to be fully defined. We present these as two related but mechanistically separate observations rather than a single statin-to-coprostanol pathway, since the available evidence does not establish that statins act specifically by upregulating bile-salt-hydrolase-mediated coprostanol formation. Accordingly, the gut microbiota is best understood not as a passive bystander in atherosclerosis, but rather as an active and potentially druggable node within the disease process.

### 1.3. Problem Statement: The Residual Risk Gap

Statins remain, and will likely remain for the foreseeable future, the cornerstone of lipid-lowering therapy, as they reliably reduce LDL-C and confer substantial reductions in vascular events [1]. Despite achieving optimal LDL-C control, a clinically meaningful proportion of patients continues to experience ischemic events. A key contributor to this residual risk is persistent low-grade systemic inflammation and oxidative stress, which are not fully normalized by conventional lipid-lowering therapy alone. These processes were never targeted by traditional lipid-lowering therapy and remain independently associated with long-term cardiovascular outcomes in metabolic-syndrome cohorts followed for over a decade [2,8].

This convergence of unresolved inflammatory, oxidative, and gut-microbial risk describes a genuine and, we would argue, evidence-based gap in current practice, as no adjunctive therapy in routine clinical use is specifically designed to act simultaneously on gut-barrier integrity, microbial metabolite production, and vascular redox signaling in patients with atherosclerosis. Probiotics and phytoantioxidants each address discrete pieces of that gap in preclinical and early clinical work. However, their combined application to atherosclerosis has not, to our knowledge, been synthesized into a coherent therapeutic framework.

### 1.4. Objective and Scope of This Review

This review evaluates the mechanistic and clinical rationale for combining probiotics and phytoantioxidants as a candidate adjunctive strategy for atherosclerosis. We explicitly distinguish claims supported by substantive evidence from those that remain hypothesis generating. Particular attention is given to four converging mechanisms: the TMA-TMAO axis, SCFA signaling, bile acid metabolism, and gut-barrier integrity. Furthermore, we detail the emerging evidence for phytoantioxidant microbiota interaction, integrating sex as an explicit biological variable throughout our evaluation.

## 2. Materials and Methods

### 2.1. Review Type and Rationale

This article is a structured narrative review rather than a systematic review or meta-analysis. We address a highly focused clinical question: whether combining probiotics with phytoantioxidants offers a mechanistically coherent adjunct to statin therapy, making a narrative evaluation the most appropriate methodological choice. Our methodology is reported according to the Scale for the Assessment of Narrative Review Articles (SANRA) [9], with a completed, item-by-item self-assessment provided as Supplementary S1.

### 2.2. Search Strategy and Selection Criteria

We searched PubMed, Embase, Web of Science, and Google Scholar for the literature published between January 2015 and August 2026. Searches combined terms describing the interventions of interest (“probiotics,” “synbiotics,” “phytoantioxidants,” “polyphenols,” “resveratrol,” “quercetin,” “curcumin,” and “chlorogenic acid”), the condition (“atherosclerosis” and “gut-vascular axis”), and the mechanistic pathways of interest (“trimethylamine-N-oxide,” “TMAO,” “short-chain fatty acids,” “bile acid metabolism,” “flavonoid intake,” and “sex differences”), using AND/OR logic adapted to each platform’s search interface and capabilities.

Within these results, we prioritized peer-reviewed randomized controlled trials, prospective cohort studies, and high-quality preclinical mechanistic studies addressing probiotic or phytoantioxidant modulation of the gut–vascular axis in atherosclerosis, supplemented with relevant systematic reviews and meta-analyses. We excluded case reports, conference abstracts without an accompanying peer-reviewed full text, non-peer-reviewed preprints, and non-English-language publications.

The corresponding author (Y.C.) screened titles and abstracts against these criteria, the co-authors reviewed the final reference list and evidence grading for completeness and relevance, and any disagreement on inclusion was resolved by discussion among all authors. As this is a narrative rather than a systematic review, we did not generate a PRISMA-style flow diagram with fixed record counts. Methodological transparency is instead provided through the completed SANRA self-assessment (Supplementary S1) and the study-level risk-of-bias appraisal of every primary source (Supplementary S2). Searches continued through 28 August 2026 to incorporate the literature raised during peer review.

### 2.3. Risk of Bias Assessment

We assessed the risk of bias for every primary study. Randomized controlled trials were evaluated using the RoB 2 tool, observational and imaging studies using an adapted ROBINS I framework, and preclinical models using the SYRCLE tool. Secondary reviews, including systematic reviews and meta-analyses, were excluded from this specific appraisal because these tools are designed exclusively for primary studies. The comprehensive assessment is available as Supplementary S2, which directly informs how we weight the subsequent evidence. Notably, one randomized trial demonstrates a high risk-of-bias due to its open label, unblinded design.

## 3. Pathophysiology: Molecular Drivers of Atherogenesis

### 3.1. Molecular Drivers of Atherogenesis

Endothelial dysfunction is the earliest and, arguably, the most consequential event in atherogenesis, defined principally by reduced nitric oxide (NO) bioavailability [1]. Under conditions of excessive reactive oxygen species (ROS) production, endothelial nitric oxide synthase (eNOS) becomes uncoupled and generates superoxide rather than NO; the resulting peroxynitrite-formation compounds vascular oxidative injury [1]. This redox imbalance in turn activates the nucleotide-binding oligomerization domain, leucine-rich repeat and pyrin domain-containing 3 (NLRP3) inflammasome, driving caspase-1 activation and maturation of interleukin-1β (IL-1β), and thereby amplifying inflammatory signaling within the vessel wall [2].

In parallel, low-density lipoprotein undergoes oxidative modification to oxidized LDL (oxLDL), which is internalized by macrophages via scavenger receptors, including cluster of differentiation 36 (CD36) and scavenger receptor-A (SR-A), converting macrophages into foam cells and producing the fatty streaks that constitute the earliest visible lesions of atherosclerosis. Lineage-tracing evidence has also updated this macrophage-centric view: vascular smooth-muscle cells transdifferentiate into foam cells as well, and, in some lesions, they outnumber those of macrophage origin [10]. Elevated lipoprotein(a) [Lp(a)] shows a graded, independent association with vulnerable-plaque features in contemporary intravascular-imaging cohorts. This pattern is distinct from overall coronary plaque burden, which tracks more closely with LDL-C and non-HDL-C, thereby implicating Lp(a) as an atherogenic lipid driver that compounds local oxidative injury [11]. Additionally, neutrophils and neutrophil extracellular traps (NETs) are increasingly recognized as active contributors to plaque destabilization, with NET components binding vascular smooth-muscle and endothelial membranes to promote lytic injury and plaque vulnerability [12]. Furthermore, gut-derived TMAO has been mechanistically linked to platelet hyperreactivity and accelerated foam-cell formation, offering a plausible bridge between microbial metabolism, innate immune activation, and thrombosis along the gut–vascular axis [13]. Endothelial dysfunction, lipoprotein oxidation, inflammasome activation, and innate-immune plaque injury thus converge on a common molecular substrate, one onto which gut-microbial and dietary-antioxidant interventions may plausibly act.

### 3.2. Sex-Specific Differences in Gut Microbiota and Atherosclerosis

Sex fundamentally modifies both gut microbial composition and the progression of atherosclerosis. A recent scoping and narrative review of coronary artery disease (CAD) summarized sex-associated differences in gut microbial composition, reporting that men often harbor relatively higher abundances of taxa such as Prevotella and certain Clostridia lineages, whereas women may be enriched in taxa such as Barnesiella and Bifidobacteriales. The same review noted that several cohorts reported stronger associations between circulating TMAO and atherosclerotic outcomes in men than in women, although substantial heterogeneity in cohort composition, sequencing methods, diet, kidney function, medication use, and statistical adjustment limits generalization [14].

Furthermore, the therapeutic response diverges by sex. In a human induced pluripotent stem cell (iPSC)-derived endothelial dysfunction model stimulated with tumor necrosis factor alpha (TNF-α) to induce a pro-atherogenic phenotype and subsequently treated with resveratrol, resveratrol produced sex-differentiated effects on mitochondrial function and inflammatory signaling. Cells derived from male donors showed greater attenuation of mitochondrial superoxide production and inflammatory cytokine release, whereas cells from female donors exhibited impaired mitochondrial respiration and reduced IL-10 signaling. These findings generate the hypothesis that baseline sex-specific mitochondrial and inflammatory phenotypes may influence responses to phytochemicals, but they do not yet establish differential clinical efficacy in atherosclerosis [15].

These findings yield two pivotal conclusions. First, sex must serve as a pre-specified stratification variable in future probiotic, phytoantioxidant, or combination trials. A formulation validated in male cohorts or animal models cannot be presumptively translated to female physiology. Second, because baseline gut microbial architecture differs by sex, the therapeutic effects of probiotic interventions may differ between male and female patients and should therefore be prospectively evaluated in sex-stratified analyses.

## 4. Probiotics: Molecular Mechanisms in Vascular Protection

Probiotics exert vascular protection through several convergent pathways, including the restoration of gut-barrier integrity, the generation of SCFAs, the modulation of bile acid metabolism, and the suppression of TMA production. Each mechanism is addressed in turn, with explicit distinction made throughout between claims supported by human clinical trials and those that remain preclinical [16].

### 4.1. Restoration of Gut-Barrier Integrity

The intestinal epithelium is the body’s first line of defense against systemic translocation of pro-inflammatory microbial products. In a murine model of enteropathogenic infection, a multi-strain probiotic formulation preserved intestinal epithelial tight-junction integrity, evidenced by sustained expression of the tight-junction protein zonula occludens-1, and prevented upregulation of plasmalemma vesicle-associated protein-1, a marker of gut–vascular-barrier disruption, thereby limiting bacterial translocation [17]. This matters mechanistically because circulating lipopolysaccharide is itself a potent activator of Toll-like-receptor-mediated endothelial inflammation: reinforcing this barrier is an upstream intervention that precedes, and may therefore help forestall, several of the downstream inflammatory events described in Section 3.

### 4.2. Short-Chain Fatty Acid Signaling

A central mechanism of probiotic action is microbial fermentation of dietary fiber into SCFAs, principally acetate, propionate, and butyrate. In the broader cardio–gut axis literature, SCFAs act through protein coupled receptors 41 and 43 (GPR41 and GPR43) on endothelial and immune cells and can inhibit NF-κB activation; these mechanisms have been documented in conditions such as heart failure, where SCFA depletion tracks with worse cardiac and inflammatory status [18]. Although direct atherosclerosis outcome trials of SCFA supplementation remain limited, these pathways converge on vascular cell adhesion molecule 1 (VCAM-1) and intercellular adhesion molecule 1 (ICAM-1) mediated leukocyte adhesion and NF-κB–driven inflammatory programs that are central to atherogenesis, supporting their mechanistic relevance as a hypothesis.

### 4.3. Bile Acid Metabolism

Probiotic strains expressing bile salt hydrolase (BSH) activity actively deconjugate primary bile acids within the intestinal lumen. A recent mechanistic study demonstrated that a BSH recombinant *Lactobacillus* strain lowered serum cholesterol in hypercholesterolemic mice through multiple coordinated pathways. Specifically, this intervention increased the abundance of *Bifidobacterium pseudolongum*, altered the composition of the bile acid pool, and derepressed hepatic cholesterol 7 alpha hydroxylase (CYP7A1) expression via the suppression of ileal farnesoid X receptor and fibroblast growth factor 15 (FXR-FGF15) signaling, thereby accelerating cholesterol catabolism into bile acids [19]. This physiological mechanism operates independently of, and serves as a potential additive complement to, statin-mediated HMG-CoA reductase inhibition. Whereas statins primarily suppress hepatic cholesterol synthesis upstream via enzymatic blockade, this gut derived pathway accelerates whole body cholesterol clearance and fecal excretion through enhanced bile acid turnover, providing a compelling pharmacological rationale for a dual route therapeutic strategy in managing refractory dyslipidemia.

The FXR and FGF15 axis identified in murine models features a direct human counterpart. Specifically, FGF15 serves as the rodent ortholog of human fibroblast growth factor 19 (FGF19). Furthermore, hepatic FXR, FGF19, and fibroblast growth factor receptor 4 (FGFR4) signaling suppresses cholesterol 7 alpha-hydroxylase (CYP7A1) mediated bile acid synthesis in humans. These mechanisms collectively support the translational relevance of probiotic bile salt hydrolase (BSH) activity for human bile acid and lipid regulation. Beyond the FXR, secondary bile acids generated by microbial 7 alpha-dehydroxylation also signal through the G protein-coupled bile acid receptor TGR5, which is expressed on macrophages, endothelial cells, and enteroendocrine cells. TGR5 activation suppresses NF-κB driven cytokine release and promotes glucagon-like peptide-1 secretion. These pathways provide an anti-inflammatory and endothelium-protective mechanism relevant to atherosclerosis and broader cardiometabolic remodeling, particularly within the context of the gut–heart axis in diabetic heart failure [20,21]. Experimental work using diabetic atherosclerosis models indicates that the related bile acid ursodeoxycholic acid (UDCA) inhibits receptor for advanced glycation end-products (RAGE) signaling, endoplasmic reticulum stress, downstream NF-κB activation, and ROS production in vascular endothelial cells exposed to hyperglycemia [22]. This effect occurs in part through Nrf2 upregulation. However, this finding reflects the pharmacological administration of an exogenous bile acid derivative rather than the action of endogenous, microbiota-generated secondary bile acids. This distinction warrants careful consideration when extrapolating these findings to probiotic-mediated bile acid remodeling. A prospective study of patients undergoing coronary angiography showed that total circulating bile acid concentrations were approximately 50% lower in individuals with CAD compared to those without the condition. A subsequent systematic review of four observational studies confirmed these consistently lower circulating bile acid levels across multiple cohorts, while cautioning that all available evidence remains strictly observational [23,24]. Nevertheless, secondary bile acids should not be regarded as uniformly protective because FXR-TGR5 signaling can produce divergent, context-dependent effects depending on which specific bile acid species predominates and on the tissue involved [25].

### 4.4. Modulation of the TMA–TMAO Axis

TMAO is a potent proatherogenic metabolite generated when gut microbial TMA, produced from dietary choline, carnitine, and phosphatidylcholine, is oxidized hepatically by flavin containing monooxygenase 3. Elevated circulating TMAO promotes macrophage foam cell formation, enhances platelet hyperreactivity, and accelerates endothelial dysfunction driving atherosclerosis [26].

A recent preclinical study showed that an orally administered nanofunctionalized probiotic construct, polydopamine coated *Lacticaseibacillus rhamnosus* GG conjugated to a ROS responsive nanoparticle, suppressed TMA production and absorption, lowered plasma TMAO, and attenuated aortic plaque formation in ApoE deficient mice [13]. The proposed causal chain is well delineated at the preclinical level: probiotic remodeling of gut microbial composition suppresses TMA generation, reduced hepatic conversion to TMAO lowers systemic proatherogenic and prothrombotic signaling, and this in turn limits macrophage lipid uptake and endothelial activation. Nevertheless, confirmatory human outcome data for this specific construct do not yet exist. A randomized double-blind trial administered a combined capsule containing 1 × 10^9^ colony-forming units (CFU) of *Lactobacillus plantarum* GLP3 with 75 mg of trans resveratrol, red wine extract, and prebiotic inulin twice daily for 12 weeks to men with established atherosclerotic cardiovascular disease, and found a significant reduction in plasma TMAO in the treated group, dropping significantly from a median of 284 to 202.5 µg/L [27]. Although the trial lacked a single agent comparator arm, preventing a definitive attribution to the probiotic component alone or proof of synergistic superiority, it nonetheless substantiates the broader premise that gut microbiota modulation successfully lowers TMAO in patients with established atherosclerosis.

### 4.5. Clinical Evidence for Probiotic Modulation of the Gut–Vascular Axis

Clinical trial evidence for probiotics in CAD remains modest in scale but is accumulating. A recent systematic review and meta-analysis of randomized trials in CAD found that probiotic monotherapy improved LDL-C and that combination probiotic regimens improved HDL-C, with more variable effects on inflammatory and oxidative markers across the pooled trials [28]. Consistent with this, a double-blind, placebo-controlled trial of *Lactobacillus rhamnosus* GG (1.6 × 10^9^ CFU/day for 12 weeks) in 44 patients with established CAD, layered onto a calorie-restricted diet in both arms, produced significant reductions in circulating lipopolysaccharide (LPS) and IL-1β relative to placebo, establishing a direct clinical correlate of the gut-barrier mechanisms described above [29].

Whether adjunctive probiotics provide measurable benefits alongside standard statin therapy remains scarcely investigated. A systematic review and meta-analysis of probiotic and statin combination therapy identified five studies (*n* = 1034), with only two randomized trials directly comparing combination therapy against statin monotherapy (*n* = 113 combined) [30]. In the sole trial reporting LDL-C outcomes (Tian et al., *n* = 49), adjunctive probiotics yielded no significant additional reduction (mean difference 0.30 mmol/L, 95% confidence interval −0.37 to 0.97). The second trial (Sun et al., *n* = 60) omitted LDL-C parameters but reported numerically fewer adverse events with probiotics (15.7% versus 25%). Significant decreases in tumor necrosis factor-α (TNF-α) and malondialdehyde derived exclusively from separate monotherapy trials lacking background statin use, which the source review classifies as indirect supporting data [30].

Furthermore, TMAO findings were sparse, heterogeneous, and unpooled. Consequently, current evidence remains preliminary, relying on isolated small-scale trials with GRADE-rated certainty classified as very low for all endpoints. Overall, available findings indicate that probiotics may contribute modestly to inflammatory and oxidative markers rather than driving further LDL-C lowering on a statin background.

### 4.6. Strain-Specific Evidence and Consensus Considerations

The probiotic literature spans multiple genera and, within genera, multiple named strains. Strain identity materially affects outcomes because gut-barrier integrity, SCFA production, bile acid metabolism, and TMAO reduction are strain-specific phenotypes that cannot be assumed to generalize across a species [17]. Strains used in atherosclerosis research include *Lactobacillus plantarum* ZDY04, which has been shown in choline-fed apolipoprotein E deficient mice to lower TMAO and reduce aortic plaque burden by remodeling specific gut bacterial taxa (including Lachnospiraceae and Erysipelotrichaceae) rather than by directly suppressing TMA-generating lyase activity or hepatic flavin-containing monooxygenase 3 (FMO3) expression [31]; *Lactobacillus reuteri* NCIMB 30242, which reduced LDL-C by 11.6% and total cholesterol by 9.1% in a nine-week randomized trial of 127 hypercholesterolemic adults, without affecting high density lipoprotein cholesterol (HDL-C) or triglycerides [32]; *Lactobacillus plantarum* 299v, which improved flow mediated dilation and reduced circulating IL-8 and IL-12 in men with stable CAD [33]; and *Lactobacillus rhamnosus* GG, which increased Akkermansia abundance and reduced LDL-C through altered ketone body synthesis in apolipoprotein E deficient mice fed a high-fat diet [34].

No consensus yet exists on which strain, dose, or duration is optimal for atherosclerosis specifically; published trials range from six to twelve weeks, and CFU dosing spans more than an order of magnitude. This absence of a validated strain selection framework, rather than any single missing trial, is the principal barrier to a defined clinical recommendation; strain comparison and dose ranging trials are an explicit priority for future research (Table 1).

**Table 1:** Representative probiotic agents, dosing regimens, and reported vascular and gut-barrier outcomes within the gut–vascular axis, stratified by preclinical and clinical evidence tiers.

Probiotic Agent/Strain	Dosage	Duration	Indication/Model	Reported Outcome(s)	Ref.	Evidence Level/RoB
Preclinical/Mechanistic Evidence						
*Multi-strain consortium:* *L. rhamnosus LR32*, *B. longum BB536*, *B. lactis BL04* (gut barrier-restorative strains)	~10^6^–10^9^ CFU (preclinical dose range)	10-day pretreatment; assessed 6–16 h post-infection	Preclinical murine model of enteropathogenic (*Salmonella typhimurium*) infection	Preserved mucus layer and ZO-1 tight-junction expression; prevented PV1 upregulation (gut–vascular barrier preservation); reduced bacterial translocation	[17]	Preclinical (murine model)
BSH-active *Lactobacillus* (recombinant strain YB334) with *Bifidobacterium pseudolongum*	Preclinical oral administration (human-equivalent CFU/day not reported)	~6–7 weeks	High-cholesterol-diet hypercholesterolemic mice	Increased *B. pseudolongum* abundance; suppressed ileal FXR–FGF15; de-repressed hepatic CYP7A1; lowered serum cholesterol	[19]	Preclinical (mouse model)
Nano-functionalized probiotic (PDMF@LGG: polydopamine-coated *L. rhamnosus* GG + ROS-responsive nanoparticle)	Preclinical construct; strain- and carrier-dependent dosing	Preclinical (ApoE^−/−^ mouse model)	Coronary and aortic atherosclerosis, ApoE-deficient mice	Suppressed TMA-producing taxa; reduced plasma TMAO; attenuated aortic plaque formation	[13]	Preclinical (ApoE^−/−^ mouse model)
Clinical Evidence						
*Lactobacillus rhamnosus* GG (single-strain, human RCT)	1.6 × 10^9^ CFU/day	12 weeks	Established coronary artery disease (double-blind RCT, *n* = 44)	Reduced circulating LPS and IL-1β vs. placebo, alongside a calorie-restricted diet	[29]	Clinical: RCT, *n* = 44; RoB: Low
*Lactobacillus plantarum* 299v (single-strain, human study)	2 × 10^10^ CFU/day	6 weeks	Men with stable coronary artery disease (nonrandomized pilot, *n* = 20)	Improved flow-mediated dilation (*p* = 0.008); reduced circulating IL-8 (*p* = 0.01) and IL-12 (*p* = 0.02); no significant change in plasma TMAO (*p* = 0.27)	[33]	Clinical: nonrandomized pilot, *n* = 20; RoB: High (no control arm)
*Lactobacillus reuteri* NCIMB 30242 (single-strain, human RCT)	2.9 × 10^9^ CFU/capsule, 2×/day	9 weeks	Hypercholesterolemic adults (double-blind, placebo-controlled RCT, *n* = 127)	Reduced LDL-C (−11.6%) and total cholesterol (−9.1%); no significant change in HDL-C or triglycerides	[32]	Clinical: RCT, *n* = 127; RoB: Some concerns (per Supplementary S2)
Multi-strain consortium (8 species: B. bifidum W23, B. lactis W51/W52, L. acidophilus W37, L. brevis W63, L. casei W56, L. salivarius W24, Lactococcus lactis W19/W58)	2.5 × 10^9^–1 × 10^10^ CFU/day	12 weeks	Obese postmenopausal women (double-blind, placebo-controlled RCT, *n* = 81)	Reduced systolic blood pressure and LDL-C; reduced arterial stiffness	[35]	Clinical: RCT, *n* = 81; RoB: Some concerns (per Supplementary S2)
*Lactobacillus plantarum* GLP3 + trans-resveratrol (combination nutraceutical, not single-strain monotherapy)	1 × 10^9^ CFU L. plantarum GLP3 + 75 mg trans-resveratrol per capsule (+ red wine extract, inulin), twice daily (~2 × 10^9^ CFU and 150 mg resveratrol/day)	12 weeks	Established atherosclerotic cardiovascular disease (double-blind RCT; *n* = 30 randomized, 14 probiotic/15 placebo evaluable)	Reduced plasma TMAO (284 → 202.5 µg/L; *p* = 0.044 vs. no significant change in placebo); increased relative Lactobacillus abundance	[27]	Clinical: RCT, *n* = 29 evaluable; RoB: Some concerns (per Supplementary S2)

BSH, bile salt hydrolase; CFU, colony-forming units; CYP7A1, cholesterol 7α-hydroxylase; FGF15, fibroblast growth factor 15; FXR, farnesoid X receptor; ICAM-1, intercellular adhesion molecule-1; LPS, lipopolysaccharide; PV1, plasmalemma vesicle-associated protein-1; RCT, randomized controlled trial; RoB, risk of bias; SCFA, short-chain fatty acid; TMA, trimethylamine; TMAO, trimethylamine-N-oxide; VCAM-1, vascular cell adhesion molecule-1; ZO-1, zonula occludens-1.

## 5. Phytoantioxidants: Molecular Targets in Vascular Protection

Phytoantioxidants act on the vasculature through both direct radical scavenging activity and indirect transcriptional activation of endogenous antioxidant defenses. We consider four extensively characterized compounds and dietary sources in the cardiovascular literature, namely resveratrol, quercetin, curcumin, and coffee as a major dietary source of chlorogenic acid, before turning to their shared molecular targets.

### 5.1. Resveratrol

Resveratrol, a stilbene polyphenol found in grapes and red wine, has attracted sustained interest for its cardiovascular effects. Mechanistically, it is reported to activate sirtuin 1 (SIRT1), a nicotinamide adenine dinucleotide (NAD)-dependent deacetylase linked to endothelial nitric oxide synthase (eNOS) activity, delayed endothelial senescence, and autophagy in vascular cells. Furthermore, gut microbial metabolism of resveratrol may generate secondary metabolites possessing additional vascular activity of their own [36,37].

Clinical translation of these findings, however, has been inconsistent, and we consider this inconsistency instructive rather than merely disappointing. A 2024 review of resveratrol trials in vascular disease found contrasting results across studies with no clear dose response relationship. Interventions using whole-food sources such as red wine produced measurable effects at substantially lower resveratrol content than concentrated tablet supplementation, whereas several high dose supplementation trials yielded null findings [36]. This is precisely the kind of unresolved clinical and mechanistic gap that a combination-based strategy is intended to address, particularly if gut microbial coadministration can indeed enhance conversion into more bioactive metabolites.

### 5.2. Quercetin

Quercetin, a flavonol abundant in onions and apples, exerts multi target vascular and neurovascular protection, including activation of Nrf2 and heme oxygenase 1 (HO-1) axis and preservation of blood brain barrier integrity [38]. A 2026 preclinical systematic review and meta-analysis consolidated evidence that quercetin attenuates experimental atherosclerosis across multiple animal models, which strengthens the biological plausibility of its vascular effects even in the continued absence of human atherosclerosis outcome data [39].

Additionally, quercetin’s translation to clinical cardiovascular endpoints remains constrained chiefly by poor oral bioavailability and rapid first pass metabolism, a limitation that motivates advanced nanoencapsulation and colonic release strategies.

### 5.3. Curcumin

Curcumin, the principal curcuminoid of turmeric (*Curcuma longa*), has shown measurable cardiovascular benefit in clinical evaluation to date. In 72 patients with type 2 diabetes and an ASCVD risk score, curcumin supplementation (500 mg three times daily for 12 weeks, added to conventional therapy) significantly reduced systolic and diastolic blood pressure, LDL-C, TNF-α, and malondialdehyde, while increasing HDL-C and improving overall ASCVD risk classification relative to conventional therapy alone [40]. However, because this preliminary trial lacked a placebo control and blinding, the observed biomarker changes should be read as open label findings rather than definitive evidence.

Mechanistically, curcumin is reported to inhibit MAPK and NF-κB signaling while activating Nrf2 to upregulate endogenous antioxidant enzymes. As with quercetin, poor bio-absorption is the main obstacle for its protective role in the impaired vascular endothelium of atherosclerosis.

### 5.4. Coffee

Coffee represents the primary dietary source of chlorogenic acid, a major phytoantioxidant whose relationship with coronary heart disease has been studied extensively. A 2023 systematic review and meta-analysis of 32 prospective cohort studies identified no significant overall association between total coffee consumption and coronary heart disease risk (relative risk 1.05, 95% confidence interval 0.97 to 1.14), though subgroup analyses indicated variations based on preparation method, intake level, and population [41]. While broader prospective data consistently link coffee intake to reduced all-cause and cardiovascular mortality, the modest or null pooled effect specifically for coronary heart disease demonstrates that its phytoantioxidant content does not guarantee uniform protection against atherosclerosis. Consequently, rather than functioning as a proven standalone risk reducing intervention, coffee is best regarded primarily as a dietary provider of chlorogenic acid type compounds with plausible, though not yet definitive, cardioprotective properties.

### 5.5. Cohort Evidence for Habitual Phytoantioxidant Intake and Subclinical Atherosclerosis

The compound-by-compound trial evidence above is small in scale and short in duration. Population-level cohort data offer a longer-horizon view of habitual phytoantioxidant exposure and atherosclerosis, distinct from short-term supplementation at pharmacological doses. The Multi-Ethnic Study of Atherosclerosis (MESA) followed 5599 participants from exam 1 (2000–2002) to exam 5 (2010–2011) and found that participants in the highest versus lowest quartile of habitual flavonoid intake had 26% lower odds of a low ankle-brachial index and 18% lower odds of carotid plaque. Flavonols, flavanol monomers, and anthocyanins specifically drove this benefit, and higher baseline anthocyanin intake also predicted slower carotid plaque progression. MESA found no significant association with carotid intima-media thickness or coronary artery calcification, and none of the associations differed by sex, race/ethnicity, or smoking status [42]. However, these findings cannot establish causation. Similar to observations with coffee consumption, habitual flavonoid intake likely marks a broader healthy dietary and lifestyle pattern rather than acting as a fully independent protective factor. Furthermore, intake assessments based on food-frequency questionnaires cannot rule out confounding by other nutrients present in flavonoid-rich foods. These findings also reflect habitual dietary patterns rather than concentrated short-course supplementation, which represents a critical distinction regarding dosage and systemic bioavailability. Taken together, the MESA supports vascular protection within the peripheral and carotid circulations rather than the coronary circulation. Consequently, future clinical trials should be specifically designed and statistically powered to evaluate distinct vascular beds rather than relying on a single generalized cardiovascular endpoint.

### 5.6. Convergent Molecular Targets: Nrf2, MAPK/NF-κB, and SIRT1

Despite their differing chemical structures, resveratrol, quercetin, curcumin, and coffee converge, across the individual studies discussed above, on a shared set of molecular targets: activation of the Nrf2 and antioxidant response element pathway, suppression of MAPK phosphorylation and NF-κB nuclear translocation, and, for resveratrol in particular, SIRT1 dependent deacetylation linked to delayed vascular senescence [36,37]. It is this convergence on a limited number of druggable nodes, more than any single compound’s individual profile, that provides the mechanistic rationale for combining multiple phytoantioxidants alongside probiotics that may enhance their bioavailability. Simultaneous administration of phytoantioxidants and probiotics can synergistically enhance endothelial function by coupling microbial biotransformation with targeted antioxidant signaling. This dual intervention effectively mitigates oxidative stress and chronic inflammation to restore impaired vascular endothelium in atherosclerosis (Table 2).

## 6. Potential Molecular Crosstalk Between Probiotics and Phytoantioxidants: Mechanisms, Evidence, and Limits

### 6.1. Mechanistic Basis for Crosstalk

Probiotics express glycosidase enzymes that deglycosylate complex dietary polyphenols into bioavailable aglycone forms with improved systemic absorption, directly addressing the bioavailability limitation identified for quercetin and curcumin above. Polyphenols, for their part, are reported to exert prebiotic like effects, selectively promoting *Lactobacillus* and *Bifidobacterium* species and thereby reinforcing short chain fatty acid producing and gut barrier-protective populations [43]. This bidirectional relationship offers a coherent mechanistic rationale for combination formulations.

A 2025 report addressing coronary endothelial dysfunction in irregular sleep and obesity associated cardiometabolic syndrome describes probiotics and phytoantioxidants such as curcumin, berberine, and epigallocatechin gallate as acting jointly on gut vascular endotoxemia, nitric oxide signaling, and Nrf2 mediated antioxidant defense [43]. Through these complementary pathways, combined administration of phytoantioxidants and probiotics may potentially suppress systemic inflammation, improve endothelial integrity, and mitigate early atherosclerotic changes.

### 6.2. Preliminary Direct Combination Evidence

The most direct clinical evidence for a combined probiotic and phytoantioxidant intervention is derived from a randomized trial evaluating a formulation comprising *Lactobacillus plantarum* GLP3, trans-resveratrol, red wine extract, and inulin administered twice daily for 12 weeks to men with established atherosclerotic cardiovascular disease [27]. This study demonstrated a significant reduction in plasma TMAO relative to the placebo group, representing the first randomized evaluation of a combined probiotic-phytoantioxidant product against a validated atherosclerosis-relevant biomarker in a clinical cohort.

However, this trial design precludes definitive conclusions regarding synergistic efficacy. Lacking single-agent comparator arms (i.e., probiotic-only or resveratrol-only), the study demonstrates superiority over placebo rather than establishing whether coadministration outperforms individual components. Consequently, whether the observed reduction stems from the probiotic, the resveratrol, their direct biochemical interaction, or additive effects across all components remains undetermined. While this trial provides the current clinical benchmark for combination therapy in atherosclerosis, it highlights the necessity for factorial designs incorporating both single-agent and combination arms to rigorously evaluate potential synergy.

Direct mechanistic evidence demonstrating interactive effects between probiotics and phytoantioxidants during coadministration remains limited but is expanding. An in vitro colonic fermentation study combining *Limosilactobacillus fermentum* with quercetin and/or resveratrol reported that coadministration significantly altered intestinal bacterial abundance, metabolite production, and overall antioxidant capacity compared to either agent alone [44]. A subsequent investigation extended these findings to fecal slurries from both hypertensive and healthy human adults in vitro, demonstrating comparable modulation of microbial composition and SCFA profiles across cohorts [45].

However, because these investigations were conducted ex vivo—omitting animal models of atherosclerosis and clinical cohorts—and lacked direct vascular endpoints, they primarily establish that the two intervention classes are mutually compatible rather than mechanistically inert. While this biological interaction represents a necessary condition for combination synergy, it is insufficient to confirm therapeutic efficacy in cardiovascular disease. Consequently, the prevailing evidence gap is defined less by a complete absence of clinical data than by the paucity of trials utilizing hard vascular or imaging endpoints capable of resolving true combination effects from individual agent activity.

## 7. Therapeutic Advances, Delivery Systems, and Clinical Evidence

Phytoantioxidant bioavailability remains a persistent obstacle to clinical translation, owing to poor aqueous solubility, extensive first-pass hepatic metabolism, and rapid systemic clearance. Advanced delivery platforms are being developed specifically to address this constraint. Nanoencapsulation strategies protect both probiotic organisms and polyphenolic compounds during gastrointestinal transit, improving viability and absorption [46], while lipid-based carriers and colonic release formulations enable synchronized delivery of both agents to the distal gut, the anatomical site where probiotic-polyphenol interaction is believed to occur. Specifically, stimuli-responsive hydrogel matrices and double-emulsion platforms facilitate site-specific, controlled intestinal release, preventing premature degradation and promoting stable microbial colonization [47,48]. A recent nano-functionalized probiotic construct demonstrates that formulation science and mechanistic biology are converging on shared delivery targets, though that specific work targeted the TMA-TMAO axis alone and has thus far been evaluated solely in a murine model [13].

## 8. Future Perspectives and Translational Challenges

The most promising direction for gut–vascular-axis therapeutics lies in personalized, metagenomically informed nutrition, which leverages individual metabolic profiles to predict therapeutic responses to specific probiotic strains or phytoantioxidant compounds. However, this precision nutrition paradigm remains largely aspirational, as validated clinical algorithms that reliably map baseline microbiome composition to therapeutic outcomes for combined formulations have yet to be established.

A conceptual precedent for this microbiome-informed approach emerges from the broader gut–cardiometabolic literature, where microbiome profiling has similarly been proposed to guide individualized interventions in diabetic heart failure [21]. Nevertheless, the same methodological and validation gaps persist, translating directly to gut–vascular therapies and highlighting the need for rigorous, mechanistically anchored clinical frameworks.

### 8.1. Broader Phytoantioxidant Classes, Microbiota-Derived Metabolites, and Dietary Confounding

Beyond the four compounds profiled previously, several additional phytoantioxidant classes exhibit plausible, albeit largely preclinical, anti-atherogenic activity.

Cyanidin-3-glucoside, an anthocyanin abundant in black rice, blackcurrants, and bilberries, engages in bidirectional interactions with the gut microbiota by reshaping microbial composition, while intestinal bacteria reciprocally metabolize it into protocatechuic acid, a derivative characterized by enhanced systemic bioavailability. In an 8-week study involving male apolipoprotein E-deficient (ApoE^−/−^) mice fed a high-fat, cholesterol-rich diet supplemented with cyanidin-3-glucoside (2 g/kg diet), treated animals exhibited elevated vascular expression of ATP-binding cassette transporter G1 (ABCG1) alongside reduced circulating cholesterol levels, including the atherogenic oxysterol 7-ketocholesterol, relative to the unsupplemented control group [49,50].

Olive oil phenolics have similarly demonstrated efficacy in human cell culture models. Oleuropein, tyrosol, hydroxytyrosol, and the hydroxytyrosol metabolite homovanillic alcohol were administered to human umbilical vein endothelial cells (HUVECs) at concentrations ranging from 2 to 20 μM for 24 h prior to tumor necrosis factor-alpha (TNF-α) stimulation. At 10 μM, all tested compounds, with the exception of tyrosol, attenuated the secretion of intercellular adhesion molecule-1 (ICAM-1) and vascular cell adhesion molecule-1 (VCAM-1). When applied at 25 to 100 μM to the EA.hy926 human endothelial cell line for 24 h, these same compounds protected against oxidative injury induced by hydrogen peroxide and oxLDL, although they did not significantly alter nitric oxide production or platelet aggregation [51].

Green tea catechins function primarily as free-radical scavengers and metal-ion chelators, attenuating lipid peroxidation markers and mitigating lipid metabolism disturbances associated with oxidative stress in atherosclerosis, a mechanism extensively documented across the broader tea catechin literature [52]. Furthermore, lycopene, an antioxidant abundant in tomato, evaluated in the human THP-1 monocyte/macrophage cell line at concentrations of 0.5 to 2 μM, dose-dependently reduced ROS generation and protected LDL from oxidation. It also suppressed smooth muscle cell proliferation and endothelial injury by modulating cellular cholesterol handling and inflammatory cytokine release [53]. Consistent with the aforementioned agents, evidence supporting these additional phytoantioxidant classes remains predominantly preclinical, necessitating translationally oriented clinical validation. A related and increasingly recognized concept is that the vascular activity attributed to a dietary phytoantioxidant may depend as much on its gut microbiota-derived metabolites as on the parent compound itself.

Ellagitannins, present in pomegranate, berries, and walnuts, are converted by the gut microbiota into urolithins. However, this conversion capacity is not universal, as only a subset of individuals, designated as producers, harbor the specific intestinal taxa required to generate urolithin A. In a 12-week, placebo-controlled, randomized trial in Japanese adults aged 40–65 years with impaired flow-mediated dilation (FMD; <7.0%) i who were pre-screened as urolithin A non-producers or low producers, oral urolithin A at 10 or 50 mg/day did not significantly improve FMD in the overall cohort; a non-significant trend was observed at 10 mg/day. Participants who exhibited FMD improvements had a lower baseline Bacillota/Bacteroidota ratio, linking treatment responsiveness to underlying microbiota composition even when urolithin A was administered directly [54]. This null finding contrasts with trials restricted to confirmed producers, where circulating urolithin metabolites generated following an ellagitannin-rich dietary challenge correlate with measurable enhancements in FMD [55].

Collectively, these findings highlight a broader translational hurdle for the field. Interindividual variability in how the gut microbiota metabolizes dietary polyphenols helps explain why single-compound supplementation trials, such as the aforementioned resveratrol studies, frequently yield inconsistent outcomes. Consequently, future trials evaluating phytoantioxidants, whether administered alone or combined with probiotics, would benefit from characterizing or stratifying participants based on their metabolic capacity to generate these microbial metabolites. Whether such bioactive metabolites, rather than the ingested parent compounds, mediate a substantial share of the cardiovascular benefits attributed to dietary phytoantioxidants remains an open mechanistic question directly relevant to the gut–vascular axis. A further limitation applies to observational and food-based evidence throughout this review, including the MESA flavonoid findings mentioned previously. Studies examining flavonoid-rich or flavonoid-enriched foods, rather than purified compounds, cannot readily isolate a phytoantioxidant’s independent effect from those of co-consumed nutrients, dietary fiber, and alternative bioactive compounds present in the whole food matrix. For instance, a diet naturally high in flavonoids typically differs substantially in fiber content, potassium levels, and overall dietary pattern from a low-flavonoid diet. This inherent dietary confounding limits the interpretability of food-frequency-based cohort evidence and trials utilizing flavonoid-enriched foods instead of isolated agents.

### 8.2. Regulatory and Manufacturing Considerations

Substantial regulatory and manufacturing challenges also remain, including the stability of probiotic colony forming units during storage, standardization of polyphenolic extract potency, and harmonization with evolving frameworks for combination pharmabiotic products. Advancing the field requires large, adequately powered, multicenter randomized trials evaluating combined formulations rather than the small, single agent studies currently dominating the literature.

### 8.3. Priorities for Definitive Clinical Trials

A definitive evaluation of this therapeutic rationale demands sex-stratified, placebo-controlled trials testing a strain-specified probiotic combined with a bioavailability enhanced phytoantioxidant formulation. Such trials should be powered for hard vascular or imaging endpoints rather than biomarker surrogates alone, establishing an actionable research agenda to bridge current translational gaps.

## 9. Conclusions

Atherosclerosis persists as a major clinical challenge despite optimal statin therapy, driven by inflammatory, oxidative, and gut microbial mechanisms that lipid-lowering management fails to address. This review advances the field by detailing sex as a biological variable in microbial composition and vascular response, providing a strain-specific appraisal of probiotics, and presenting a unified model of gut vascular crosstalk that maps the current boundaries of the combination hypothesis.

Probiotics and phytoantioxidants act on mechanistically distinct but functionally convergent nodes of the gut vascular axis, namely gut-barrier integrity; short-chain fatty acid and bile acid signaling; the TMA and TMAO axis; and Nrf2, MAPK, and SIRT1 dependent endothelial protection, and the preliminary single class evidence for each is genuinely encouraging. However, translating these mechanisms into therapeutic recommendations requires caution. Current clinical evidence is constrained by trials lacking single-agent comparator arms, reliance on ex vivo data, small sample sizes, and general methodological limitations.

Rigorous, sex-stratified, and strain-specified combination trials, rather than additional single class studies or narrative overviews, represent the essential next step to determine whether this dual intervention strategy can meaningfully reduce residual cardiovascular and cerebrovascular risk.

### An Integrated Model of the Gut–Vascular Axis: Mechanisms, Interventions, and Translational Gaps

Figure 1 organizes residual cardiovascular risk into a gut-microbial node and a vascular node, and maps each intervention class onto the node it predominantly targets. The proposed synergy between the two classes of probiotic-enhanced phytoantioxidant bioavailability and phytoantioxidant prebiotic-like effects remains hypothesized rather than directly demonstrated. As with every combination strategy raised in this review, the figure is a framework for future trials, not evidence of an established therapeutic pathway.

## Figures and Tables

**Figure 1:**
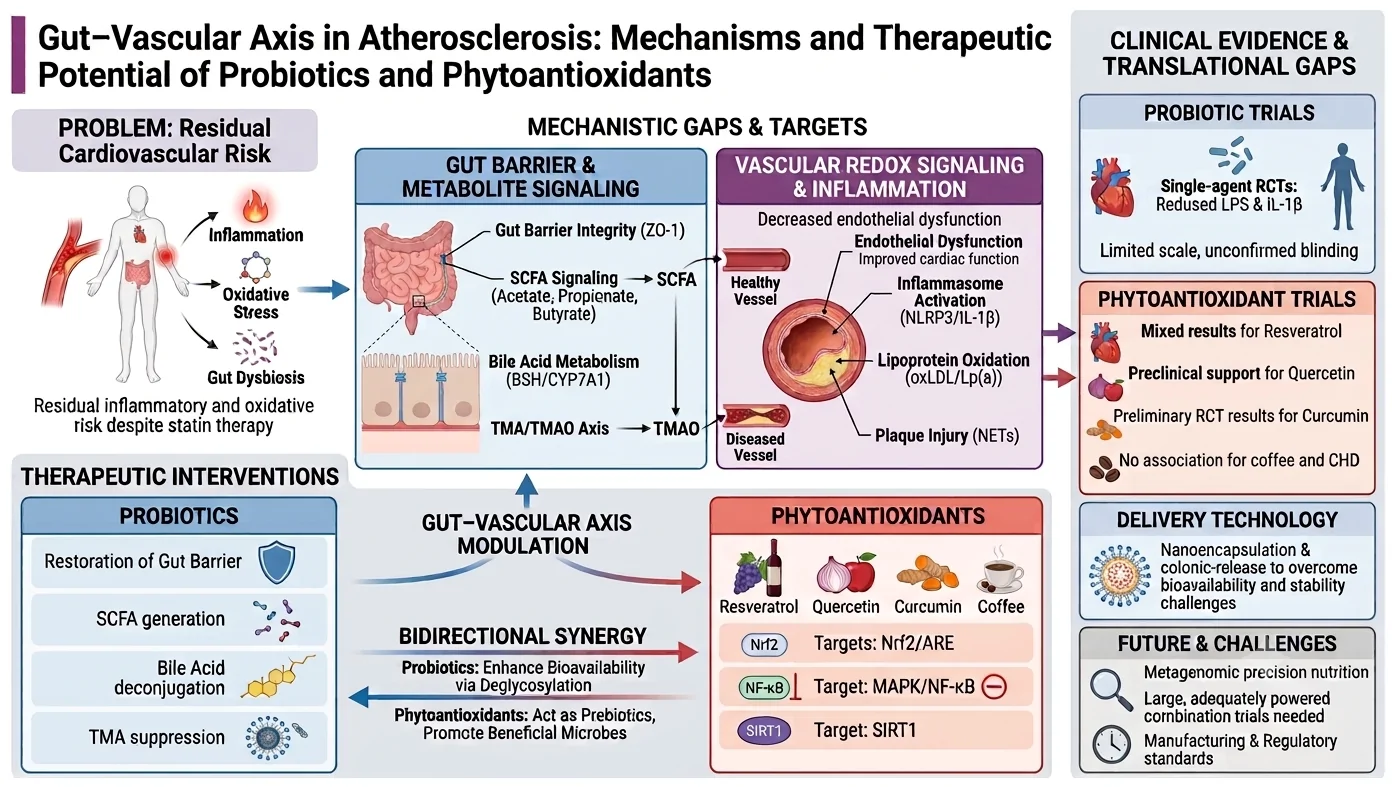
An integrated model of the gut–vascular axis in atherosclerosis. Residual inflammatory, oxidative, and gut-dysbiotic risk despite statin therapy is organized into two convergent mechanistic nodes: gut barrier and metabolite signaling (gut-barrier integrity, SCFA signaling, bile acid metabolism, and the TMA–TMAO axis) and vascular redox signaling and inflammation (endothelial dysfunction, NLRP3 inflammasome activation, lipoprotein oxidation, and NET-mediated plaque injury), each annotated with its supporting reference. Probiotics and phytoantioxidants are shown acting predominantly at the gut and vascular nodes, respectively, linked by a proposed bidirectional synergy between probiotic-mediated deglycosylation and phytoantioxidant prebiotic-like effects. The right panel summarizes the current clinical evidence and translational gaps for each intervention class, together with delivery-technology approaches and the challenges remaining for the field.

**Table 2:** Representative phytoantioxidants, dosing regimens, and reported vascular outcomes, with the level of evidence (preclinical vs. clinical, and single-trial vs. pooled).

Phytoantioxidant	Dosage	Duration	Indication/Model	Reported Outcome(s)	Ref.	Evidence Level/RoB
Preclinical/Mechanistic Evidence						
Quercetin	Not established in humans for atherosclerosis; dosing varies by preclinical model	Variable (preclinical systematic review)	Experimental atherosclerosis across multiple animal models	Consistent attenuation of atherosclerosis across preclinical models; activation of Nrf2–heme oxygenase-1 axis and blood–brain barrier preservation reported in related neurovascular literature	[38,39]	Preclinical (systematic review/meta-analysis); RoB: N/A (secondary synthesis)
Clinical Evidence						
Resveratrol	Highly variable across reviewed RCTs; whole-food sources (e.g., red wine) vs. concentrated tablet supplementation	Variable across the RCTs reviewed	Vascular disease (evidence synthesized from clinical trials)	Contrasting, non-dose-dependent clinical effects on endothelial and blood-pressure endpoints; red-wine-source interventions showed effects at lower resveratrol content than high-dose tablets, which frequently showed null findings	[36]	Clinical: narrative synthesis of multiple RCTs; RoB not pooled
Curcumin	500 mg three times daily (1500 mg/day)	12 weeks	Type 2 diabetes mellitus with elevated ASCVD risk (RCT, *n* = 72)	Reduced SBP/DBP, LDL-C, TNF-α, and MDA; increased HDL-C; improved ASCVD risk classification	[40]	Clinical: RCT, *n* = 72; RoB: High (open-label, no placebo arm)
Coffee (chlorogenic-acid-type phytoantioxidants)	Habitual consumption level, not a supplement dose	Pooled across 32 prospective cohort studies	General population; coronary heart disease	No significant overall association with CHD risk (RR 1.05, 95% CI 0.97–1.14); effects vary by preparation method and subgroup	[41]	Clinical: pooled meta-analysis, 32 cohorts; RoB not pooled
Dietary flavonoids (habitual intake)	Habitual dietary intake, estimated by food-frequency questionnaire (not a supplement dose)	Prospective cohort, exam 1 (2000–2002) to exam 5 (2010–2011)	General adult population; MESA cohort (*n* = 5599)	Higher intake associated with lower odds of low ABI and carotid plaque; no significant association with IMT or coronary artery calcium; associations did not differ by sex, race/ethnicity, or smoking status	[42]	Clinical: prospective cohort; RoB: Moderate (per Supplementary S2)

ABI, ankle-brachial index; ASCVD, atherosclerotic cardiovascular disease; CHD, coronary heart disease; CI, confidence interval; HDL-C, high-density lipoprotein cholesterol; IMT, intima-media thickness; LDL-C, low-density lipoprotein cholesterolA, malondialdehyde; MESA, Multi-Ethnic Study of Atherosclerosis; Nrf2, nuclear factor erythroid 2-related factor 2; RCT, randomized controlled trial; RoB, risk of bias; RR, relative risk; SBP/DBP, systolic/diastolic blood pressure; TNF-α, tumor necrosis factor-α.

## Data Availability

No new data were created or analyzed in this study. Data sharing is not applicable to this article.

## Supplementary Materials

The following supporting information can be downloaded at https://www.mdpi.com/article/10.3390/ijms27198535/s1. References [3,8,11,13,17,19,27,29,31,32,33,34,35,40,42] are cited in the Supplementary Material.
