# Supplementary material for: Targeting the Gut Vascular Axis in Atherosclerosis: Probiotic and Phytoantioxidant Crosstalk as a Hypothesis-Generating Framework for Residual Cardiovascular Risk
Source: Int J Mol Sci. 2026 Sep 24;27(19):8535. doi: 10.3390/ijms27198535 (PMC13664750; doi:10.3390/ijms27198535)
Supplement: Supplementary file 1 [file ijms-27-08535-s001.zip › Supplement S1 SANRA.pdf]

SANRA Self-Assessment for "Targeting the Gut Vascular Axis in Atherosclerosis: Probiotic and Phytoantioxidant Crosstalk as a Hypothesis-Generating Framework for Residual Cardiovascular Risk"

This document reports the authors' self assessment of the manuscript using SANRA, the Scale for the Assessment of Narrative Review Articles (Baethge, Goldbeck-Wood, and Mertens, 2019, Research Integrity and Peer Review, 4, 5, doi:10.1186/s41073-019-0064-8). SANRA was developed because, unlike systematic reviews, narrative reviews previously had no standard instrument for quality appraisal. It scores six domains from 0 (low standard) to 2 (high standard), for a maximum of 12. This manuscript is presented, and should be evaluated, as a narrative review that follows PRISMA informed transparency principles for search and referencing rather than as a registered systematic review; the rationale for that scope decision is given in Section 2.1 of the main manuscript.

Total score: 11 out of 12. Five of six items are scored at the maximum. Item 3, description of the literature search, is scored 1 out of 2, and the reason for that score is stated below rather than concealed.

Item	SANRA domain	Score (0 to 2)	Justification
1	Explanation of the importance of the review question for medical practice or research	2	Section 1.3 states the clinical gap directly: a substantial proportion of statin-treated patients continue to have ischemic events, and this residual risk is attributed to inflammatory, oxidative, and gut microbial mechanisms that lipid lowering therapy does not address. The importance of the topic is argued on that basis rather than asserted generically.
2	Statement of the specific aims or the questions the review addresses	2	Section 1.4 states the review's aim explicitly: to evaluate, on mechanistic and available clinical grounds, the case for combining probiotics and phytoantioxidants as an adjunctive strategy for atherosclerosis, with four named converging mechanisms.
3	Description of the literature search, including databases used, search terms, and the time frame covered	1	Section 2.2 names the platforms actually searched (PubMed, Embase, Web of Science, and Google Scholar), lists the concept-based search terms used, and states the search date range. It does not report a literal database-specific search string, an exact record count, or dual independent screening, and Section 2.1 states this limitation directly rather than implying full systematic search rigor. This is the one domain where the manuscript falls short of the maximum standard, and it does so by design and disclosure rather than by omission.
4	Referencing, meaning that the statements in the review are adequately and correctly supported by citations	2	Every reference in this manuscript was individually checked against its DOI and against the specific claim it supports, following an audit that identified and removed fabricated and mismatched citations from an earlier draft. This verification process is documented here and in the accompanying response-to-reviewers letter, which confirms independent source

			verification for every reference added during revision.
5	Scientific reasoning, meaning that evidence is presented and interpreted according to accepted scientific principles, including a clear distinction between correlation and causation and between preclinical and clinical evidence	2	The manuscript consistently labels preclinical versus clinical evidence, states dosing that has not been established in humans as such, reports a null finding for coffee and coronary heart disease rather than omitting it, and flags an open label trial design and its implication for causal interpretation in Section 5.3 and in the Supplementary Risk of Bias Assessment.
6	Appropriate presentation of data, meaning that quantitative information such as effect sizes and study numbers is presented accurately and without selective emphasis	2	Tables 1 and 2 state, for every row, whether the evidence is preclinical or clinical and whether it comes from a single trial or a pooled analysis. Reported effect sizes, including a non-significant pooled relative risk for coffee and coronary heart disease, are given with their confidence intervals rather than rounded toward significance.

Notes on the item 3 score

A full score on item 3 would require a reproducible search strategy of the kind used in a registered systematic review: explicit Boolean search strings run against named databases on a stated date, a documented total record count, and a defined screening process with more than one reviewer. This manuscript does not have those elements, and Section 2.1 of the main text says so directly rather than presenting search language that implies a systematic review was conducted. We regard this as the honest score for the search methodology actually used, and we would rather report it accurately than inflate it.

Authors intending to pursue a fully systematic evidence synthesis on this topic, rather than the narrower combination hypothesis addressed here, are directed to the recent registered scoping review cited as reference 22 in the main manuscript, which screened 3556 records across four databases to reach 192 included studies.

How this table may be used

This self assessment is offered as a supplementary file for editorial and peer review reference. It may be cited from the main manuscript, for example as “See Supplementary SANRA Self-Assessment,” and it is intended to make explicit, in advance of review, the same limitations that a careful reviewer would otherwise need to identify independently.
