# Supplementary material for: Targeting the Gut Vascular Axis in Atherosclerosis: Probiotic and Phytoantioxidant Crosstalk as a Hypothesis-Generating Framework for Residual Cardiovascular Risk
Source: Int J Mol Sci. 2026 Sep 24;27(19):8535. doi: 10.3390/ijms27198535 (PMC13664750; doi:10.3390/ijms27198535)
Supplement: Supplementary file 1 [file ijms-27-08535-s001.zip › Supplement S2- Risk of Bias.pdf]

Quality and Risk-of-Bias Assessment of the Primary Studies Cited in "Targeting the Gut Vascular Axis in Atherosclerosis: Probiotic and Phytoantioxidant Crosstalk as a Hypothesis-Generating Framework for Residual Cardiovascular Risk"

RoB 2 (randomized controlled trials) • ROBINS-I-adapted (cohort/imaging studies) • SYRCLE (preclinical/animal studies) — Supplementary methods documentation, referenced from Section 2.3 of the main manuscript.

This document applies risk-of-bias appraisal to every primary empirical study in the manuscript's verified reference list, using the tool appropriate to each study's design. Systematic reviews and meta-analyses in the reference list (Dong et al. [28,30], Chen D. et al. [39], Park et al. [41]) are excluded from this appraisal, since RoB 2, ROBINS-I, and SYRCLE are designed for primary studies rather than secondary syntheses; narrative/mechanistic reviews and in vitro-only papers in the reference list are likewise excluded for the same reason. Judgments below are based on the abstracts, results summaries, and methods excerpts retrieved during citation verification, not on a line-by-line read of each paper's full-text Methods section. Where a domain could not be confidently judged from the available material, it is marked "Unclear" rather than assigned an optimistic default — consistent with standard risk-of-bias practice, which treats missing information as a source of uncertainty, not evidence of low risk. See "Summary and Outstanding Actions" for the specific verification steps recommended before this document is finalized for submission.

A. RoB 2 — Randomized Controlled Trials (n = 5)

The reference list contains five randomized controlled trials. RoB 2 assesses five domains: D1 = Randomization process; D2 = Deviations from intended interventions; D3 = Missing outcome data; D4 = Measurement of the outcome; D5 = Selection of the reported result.

Trial	D1	D2	D3	D4	D5	Overall
Moludi et al., 2021 (Nutr. J.) [ref. 29]	Low	Low	Low	Low	Low	Low
El-Rakabawy et al., 2025 (Sci. Rep.) [ref. 40]	Some concerns	High	Unclear	Some concerns	Some concerns	High
Jones et al., 2012 (Eur. J. Clin. Nutr.) [ref. 32]	Some concerns	Low	Some concerns	Low	Some concerns	Some concerns
Szulińska et al., 2018 (Nutrients) [ref. 35]	Some concerns	Some concerns	Unclear	Low	Some concerns	Some concerns
Spasova et al., 2024 (Folia Med.) [ref. 27]	Low	Low	Some concerns	Low	Some concerns	Some concerns

Moludi et al., 2021 — Nutrition Journal [ref. 29]

Effect of probiotic supplementation along with calorie restriction on metabolic endotoxemia, and inflammation markers in coronary artery disease patients: a double-blind, placebo-controlled, randomized clinical trial.

D1 — Randomization process — Low: Full-text Methods confirm: 44 patients with CAD were allocated 1:1 to Lactobacillus rhamnosus GG capsules or matching placebo capsules; "a third party who was blind to the study gave the randomization sequence extracted from allocation software," and investigators, providers, and participants were blinded after assignment. Both sequence generation and allocation concealment are adequately described.

D2 — Deviations from intended interventions — Low: Explicitly double-blind, placebo-controlled; both arms additionally received the same calorie-restricted diet co-intervention, so no differential co-intervention is apparent.

D3 — Missing outcome data — Low: Full-text confirms an intention-to-treat analysis including all 44 randomized patients (22 per arm); the flowchart reports exactly 1 dropout in the intervention group and 2 in the placebo group, with reasons and handling both accounted for.

D4 — Measurement of the outcome — Low: Primary outcomes (serum IL-1β, TLR4, IL-10, LPS) are objective laboratory biomarkers with low susceptibility to assessor subjectivity, consistent with the double-blind design.

D5 — Selection of the reported result — Low: Full-text confirms registration in the Iranian Registry of Clinical

Trials, IRCT20121028011288N15, with local ethics approval (IR.TBZMED.REC.1397.184); circulating endotoxin (LPS) is explicitly declared the primary outcome, with inflammatory markers as secondary.

Overall judgment: Low. Upgraded from "Some concerns" after confirming the full-text Methods and IRCT registration record — this resolves the verification action item below. No domain is judged High or Some concerns.

El-Rakabawy et al., 2025 — Scientific Reports [ref. 40]

Curcumin supplementation improves the clinical outcomes of patients with diabetes and atherosclerotic cardiovascular risk.

D1 — Randomization process — Some concerns: Seventy-two patients with type 2 diabetes and elevated ASCVD risk were “randomly assigned,” but sequence generation and allocation concealment are not detailed in the available extracts.

D2 — Deviations from intended interventions — High: The comparison is curcumin (500 mg three times daily) plus conventional therapy versus conventional therapy alone — there is no placebo arm and no blinding is described. This open-label design carries a material risk of performance bias (participants and treating clinicians aware of allocation).

D3 — Missing outcome data — Unclear: Completion and attrition figures for the 72 enrolled patients are not confirmed in the available extracts.

D4 — Measurement of the outcome — Some concerns: Outcomes (SBP/DBP, LDL-C, HDL-C, TNF-α, MDA) are objective laboratory and hemodynamic measures, which limits but does not eliminate detection bias risk in the absence of blinded outcome assessment — blood-pressure measurement in particular has operator-dependent variability.

D5 — Selection of the reported result — Some concerns: Trial registration and a pre-specified statistical analysis plan are not confirmed in the available extracts.

Overall judgment: High, driven primarily by D2 (absence of blinding/placebo control). This does not mean the reported effects are false — the biomarker changes are directionally consistent with curcumin's known mechanism — but the open-label design means the effect sizes reported in Section 5.3 of the main manuscript should be described as preliminary rather than definitive, and this should be reflected explicitly in the text before submission.

Jones et al., 2012 — European Journal of Clinical Nutrition [ref. 32]

Cholesterol lowering and inhibition of sterol absorption by Lactobacillus reuteri NCIMB 30242: a randomized controlled trial.

D1 — Randomization process — Some concerns: Described as a randomized, double-blind, placebo-controlled, parallel-arm, multicentre study; 127 subjects completed a 9-week intervention comparing L. reuteri NCIMB 30242 capsules to placebo capsules. Specific sequence-generation and allocation-concealment methods are not detailed in the available extracts.

D2 — Deviations from intended interventions — Low: Explicitly double-blind and placebo-controlled (capsule vs. matched placebo); no differential co-intervention is described.

D3 — Missing outcome data — Some concerns: 127 subjects are reported to have completed the study, but the number initially randomized and reasons for any attrition are not confirmed in the available extracts.

D4 — Measurement of the outcome — Low: Primary outcomes (LDL-C, total cholesterol, non-HDL-C, apoB-100) are objective serum lipid measures with low susceptibility to assessor subjectivity.

D5 — Selection of the reported result — Some concerns: A trial registry number was not identified in the available source material; pre-specification of the primary lipid endpoint cannot be confirmed without the full-text registration record.

Overall judgment: Some concerns, driven by D1, D3, and D5 — the same pattern as the Moludi trial above. Table 1 of the main manuscript currently states "RoB: Low" for this trial; that claim should be revised to "Some concerns" to match this appraisal, or upgraded to Low only after the full-text Methods section confirms randomization sequence generation, allocation concealment, and trial registration.

Szulińska et al., 2018 — Nutrients [ref. 35]

Multispecies probiotic supplementation favorably affects vascular function and reduces arterial stiffness in obese postmenopausal women — a 12-week placebo-controlled and randomized clinical study.

D1 — Randomization process — Some concerns: Obese postmenopausal women were randomized to placebo or one of two probiotic doses over 12 weeks; specific sequence-generation and allocation-concealment methods are not detailed in the available extracts.

D2 — Deviations from intended interventions — Some concerns: Described as placebo-controlled; explicit blinding of participants and outcome assessors for this specific trial is not directly confirmed in the available extracts, though companion trials from the same research group using the same product (Ecologic® Barrier) are consistently reported as double-blind.

D3 — Missing outcome data — Unclear: Completion and attrition figures for the enrolled cohort (n = 81 per Table 1) are not confirmed in the available extracts.

D4 — Measurement of the outcome — Low: The primary endpoint (endothelium-dependent vascular function) and secondary biochemical parameters are objective, instrument-based measures.

D5 — Selection of the reported result — Some concerns: Trial registration and a pre-specified statistical analysis plan are not confirmed in the available extracts.

Overall judgment: Some concerns, driven by D1, D2, D3, and D5. As with Jones et al. above, Table 1's "RoB: Low" judgment for this trial should either be revised to "Some concerns" or confirmed against the full-text Methods and trial registration before the manuscript is finalized.

Spasova et al., 2024 — Folia Medica (Plovdiv) [ref. 27]

Effect of Lactobacillus plantarum supplementation on trimethylamine-N-oxide levels in 30 patients with atherosclerotic cardiovascular disease: a double-blind randomized controlled trial.

D1 — Randomization process — Low: Full-text Methods confirm randomization was performed centrally at the Center of Applied Studies and Innovation; probiotic and placebo were pre-packed into sequentially coded, uniquely numbered bottles at a 1:1 ratio and assigned to participants in recruitment order, providing adequate sequence generation and allocation concealment from the enrolling site.

D2 — Deviations from intended interventions — Low: Full-text confirms both researchers and participants were blinded to treatment allocation throughout the 12-week intervention; compliance was verified via returned product counts, with no differential co-intervention described.

D3 — Missing outcome data — Some concerns: Of 30 randomized patients (intended 15 per arm), the reported analysis includes 15 placebo and 14 probiotic patients (29 total); the reason for the single missing probiotic-arm participant is not stated in the available extracts.

D4 — Measurement of the outcome — Low: TMAO was quantified by triple-quadrupole tandem mass spectrometry with liquid chromatography, an objective, high-precision analytical method with low susceptibility to assessor subjectivity.

D5 — Selection of the reported result — Some concerns: Ethics approval and grant support (Bulgarian Society of Cardiology) are reported and TMAO is explicitly declared the primary outcome consistent with what is presented, but a prospective trial registry number (e.g., ClinicalTrials.gov) was not identified in the available extracts.

Overall judgment: Some concerns, driven by D3 and D5. This is a well-conducted small trial — centrally randomized with coded, concealed allocation, double-blind, and an objective analytical outcome measure — but the single unexplained dropout and the absence of a located trial registration keep it from a "Low" rating. Table 1's current note ("RoB: not yet incorporated into Supplementary File S2") should be replaced with this verdict.

B. ROBINS-I (Adapted) — Observational Cohort and Imaging-Substudy Evidence

ROBINS-I (Risk Of Bias In Non-randomized Studies of Interventions; Sterne et al., 2016) formally comprises seven

domains grouped into pre-intervention, at-intervention, and post-intervention bias. None of the four non-randomized studies in this reference list involves a discrete "intervention" in the ROBINS-I sense — they are prospective or retrospective observational analyses of biomarker/exposure–outcome associations. A simplified three-domain adaptation (confounding, selection into the study, outcome measurement) is therefore used here, which is the same simplification applied in the template example this document follows. This is a reasonable adaptation for exposure-outcome cohort designs, but it is a deviation from the full ROBINS-I instrument and should be flagged as such if this table is reused in a formal risk-of-bias appendix.

Study	Confounding	Selection	Outcome measurement	Overall
Saarinen et al., 2024 (BMC Cardiovasc. Disord.) [ref. 8]	Moderate	Low	Low	Moderate
Zhang et al., 2025/26 (Front. Neurol.) [ref. 3]	Moderate	Moderate	Low	Moderate
Erlinge et al., 2025 (JACC, PROSPECT II) [ref. 11]	Unclear	Low	Low	Unclear
Bondonno et al., 2024 (ATVB, MESA) [ref. 42]	Moderate	Low	Low	Moderate

Saarinen et al., 2024 — BMC Cardiovascular Disorders [ref. 8]

The association between inflammation, arterial stiffness, oxidized LDL and cardiovascular disease in Finnish men with metabolic syndrome — a 15-year follow-up study.

Confounding — Moderate: Single prospective cohort of 105 Finnish men with metabolic syndrome followed a mean of 16.4 years; the design evaluates whether baseline biomarkers/risk scores predict later cardiovascular events within this cohort, rather than comparing an exposed to an unexposed group, which limits how directly classical confounding-adjustment domains apply.

Selection — Low: Consecutive recruitment within a dedicated regional metabolic-syndrome research program, with a long, well-documented follow-up period.

Outcome measurement — Low: Composite hard outcomes (myocardial infarction, stroke, revascularization, death) were retrieved from electronic medical records using consistent ascertainment criteria across the cohort.

Overall: Moderate. External-validity note (not a risk-of-bias domain per se): the cohort is exclusively male and single-country, which limits generalizability of the specific effect sizes to other populations — this should be stated as a limitation wherever this study's findings are used to support a general claim in the main text.

Zhang et al., 2025/2026 — Frontiers in Neurology [ref. 3]

Prediction of stroke recurrence for patients with middle cerebral artery atherosclerotic disease by CT perfusion.

Confounding — Moderate: Retrospective, four-center design with multivariate logistic regression adjustment reported for stenosis severity and other covariates; the complete covariate list and possible confounding by treatment intensity between recurrence and non-recurrence groups are not confirmed in the available extracts.

Selection — Moderate: Patients were included from four stroke centers with defined post hoc exclusion criteria (poor follow-up data, contralateral stenosis, moyamoya syndrome, poor image quality); the criteria appear clinically justified and pre-specified, but post hoc exclusion in a retrospective design always carries some selection-bias risk.

Outcome measurement — Low: Stroke recurrence is an objective clinical endpoint ascertained over a defined 3-month follow-up window, with the CT-perfusion exposure (Tmax volumes) measured using a standardized imaging protocol.

Overall: Moderate.

Erlinge et al., 2025 — Journal of the American College of Cardiology (PROSPECT II substudy) [ref. 11]

Lipoprotein(a), cholesterol, triglyceride levels, and vulnerable coronary plaques: a PROSPECT II substudy.

Confounding — Unclear: This is a substudy of the previously published PROSPECT II prospective imaging cohort; the specific covariate-adjustment strategy used for the Lp(a)–plaque association analyzed here is not

confirmed in the available extracts (title/abstract-level access only).

Selection — Low: As a nested substudy of a cohort defined at enrollment into the parent PROSPECT II trial, selection into this analysis is unlikely to be materially biased relative to the parent cohort.

Outcome measurement — Low: Plaque characteristics were assessed using protocol-driven intravascular imaging (IVUS/OCT/NIRS, per the parent trial's standard methodology), which is an objective, technician-adjudicated measure.

Overall: Unclear. This judgment is driven by limited available detail rather than an identified flaw — the full-text Methods section should be reviewed directly before this study is used to support a specific quantitative claim.

Bondonno et al., 2024 — Arteriosclerosis, Thrombosis, and Vascular Biology (MESA) [ref. 42]

Associations Between Flavonoid Intake and Subclinical Atherosclerosis: The Multi-Ethnic Study of Atherosclerosis.

Confounding — Moderate: Repeated-measures models across MESA exam 1 (2000–2002) and exam 5 (2010–2011) were multivariable-adjusted, with stratified analyses and interaction terms exploring effect modification by time, sex, race/ethnicity, and smoking status; the complete covariate-adjustment set (e.g., whether traditional lipid, blood-pressure, and total energy-intake covariates were all included) is not detailed in the available extracts.

Selection — Low: Participants (n = 5599) were drawn from the well-characterized, prospective MESA cohort with exposure (validated food-frequency questionnaire) and outcome data collected at defined study exams; selection into this analysis mirrors the parent cohort's established recruitment.

Outcome measurement — Low: Ankle-brachial index, carotid ultrasound (plaque presence and intima-media thickness), and CT-based coronary artery calcification are objective, protocol-driven measures consistent with MESA's established imaging methodology.

Overall: Moderate, driven by incomplete confounding-adjustment detail rather than an identified design flaw — comparable to the Saarinen et al. entry above. Table 2's current note ("RoB: not yet incorporated into Supplementary File S2") should be replaced with this verdict.

C. SYRCLE's RoB Tool — Preclinical (Animal) In Vivo Studies

SYRCLE's Risk of Bias tool (Hooijmans et al., 2014) adapts the Cochrane RoB framework for animal intervention studies, covering selection bias (sequence generation, baseline characteristics, allocation concealment), performance bias (random housing, blinding of caregivers/investigators), detection bias (random outcome assessment, blinded outcome assessors), and attrition/reporting bias (complete outcome data, selective reporting).

As in the template this document follows, the high prevalence of "Unclear" judgments below is expected and normal for preclinical literature, where randomization and blinding are frequently performed but not explicitly described in the abstract or results summary available for this appraisal.

Preclinical study	Selection	Performance	Detection	Attrition/Reporting
Naso et al., 2025 (Front. Microbiol.) [ref. 17]	Unclear	Low	Unclear	Low
Liu et al., 2025 (Nutrients) [ref. 19]	Unclear	Unclear	Unclear	Low
Chen et al., 2025 (Nat. Commun.) [ref. 13]	Unclear	Low	Unclear	Low
Zhai et al., 2022 (Appl. Microbiol. Biotechnol.) [ref. 34]	Unclear	Unclear	Unclear	Low
Qiu et al., 2018 (Food Funct.) [ref. 31]	Unclear	Low	Unclear	Low

Naso et al., 2025 — Frontiers in Microbiology [ref. 17]

A multi-strain probiotic formulation preserves intestinal epithelial and vascular barriers during enteropathogenic infection.

Selection — Unclear: Group allocation method for C57BL/6 mice (random vs. sequential cage assignment to control/infected/treatment groups) is not specified in the available extracts.

Performance — Low: A standardized 10-day multi-strain probiotic pretreatment protocol and a consistent Salmonella typhimurium infection challenge were applied uniformly across the described groups.

Detection — Unclear: Blinding of investigators performing histological and immunofluorescence quantification (mucus layer, ZO-1, PV1) is not specified in the available extracts.

Attrition/Reporting — Low: Outcomes are reported completely across all three described groups (control, infected, treatment) with no apparent selective reporting.

Overall: Unclear.

Liu et al., 2025 — Nutrients [ref. 19]

Cholesterol-lowering mechanism of Lactobacillus bile salt hydrolase through regulation of Bifidobacterium pseudolongum in the gut microbiota.

Selection — Unclear: Allocation method for mice to control versus BSH-recombinant-strain (YB334) groups is not specified in the available extracts.

Performance — Unclear: A defined high-cholesterol-diet protocol was used, but standardization of housing conditions across groups is not explicitly confirmed.

Detection — Unclear: Blinding of biochemical (serum cholesterol) and metagenomic-sequencing analysis is not specified in the available extracts.

Attrition/Reporting — Low: Cholesterol parameters and gut-microbiota compositional outcomes are reported comprehensively for the described experimental groups.

Overall: Unclear.

Chen et al., 2025 — Nature Communications [ref. 13]

Nano-functionalized probiotic treats atherosclerosis via inhibiting intestinal microbiota-TMA-TMAO axis.

Selection — Unclear: Randomization of ApoE−/− mice to treatment arms is not explicitly confirmed in the available extracts, although Nature Communications' standard reporting requirements make undisclosed randomization less likely than in a lower-tier venue — this is a plausibility judgment, not a confirmed methods detail.

Performance — Low: A standardized ApoE−/− atherosclerosis model and a single, well-defined nanoparticle–probiotic construct (PDMF@LGG) were administered uniformly to the treatment arm.

Detection — Unclear: Blinded assessment of aortic plaque burden and plasma TMAO quantification is not confirmed in the available extracts.

Attrition/Reporting — Low: TMAO suppression, plaque attenuation, and microbiota shifts are reported comprehensively across the described treatment and control arms.

Overall: Unclear.

Zhai et al., 2022 — Applied Microbiology and Biotechnology [ref. 34]

Lactobacillus rhamnosus GG protects against atherosclerosis by improving ketone body synthesis.

Selection — Unclear: Allocation of ApoE−/− mice to LGG-treated versus control groups is not specified in the available extracts.

Performance — Unclear: A defined LGG gavage protocol in an ApoE−/− model is described, but standardization of housing conditions across groups is not explicitly confirmed in the available extracts.

Detection — Unclear: Blinded assessment of ketone-body synthesis markers and atherosclerotic lesion burden is not confirmed in the available extracts.

Attrition/Reporting — Low: Metabolic and atherosclerosis outcomes are reported comprehensively across the described groups.

Overall: Unclear.

Qiu et al., 2018 — Food & Function [ref. 31]

Lactobacillus plantarum ZDY04 exhibits a strain-specific property of lowering TMAO via the modulation of gut microbiota in mice.

Selection — Unclear: Allocation of choline-fed ApoE−/− mice to ZDY04-treated versus control groups is not specified in the available extracts.

Performance — Low: A standardized 1.3% choline-feeding protocol and a single, well-defined bacterial strain (ZDY04) were administered uniformly to the treatment arm.

Detection — Unclear: Blinded quantification of plasma TMAO and gut-microbiota composition is not confirmed in the available extracts.

Attrition/Reporting — Low: TMAO and microbiota outcomes are reported comprehensively across the described groups.

Overall: Unclear.

D. Non-Comparative Pilot Study — Not Formally Assessable by RoB 2, ROBINS-I, or SYRCLE

One reference in the primary-study list — Malik et al. [ref. 33] — does not fit any of the three tools above. It is neither randomized (ruling out RoB 2) nor a comparative observational design with a distinct comparator group (ruling out ROBINS-I) nor an animal model (ruling out SYRCLE): it is a single-arm, pre-post pilot study.

Malik et al., 2018 — Circulation Research [ref. 33]

Lactobacillus plantarum 299v supplementation improves vascular endothelial function and reduces inflammatory biomarkers in men with stable coronary artery disease.

Design — High risk of bias: Twenty men with stable CAD consumed a single daily probiotic drink (20 billion CFU) for 6 weeks; outcomes (brachial flow-mediated dilation, plasma inflammatory biomarkers) were compared before versus after supplementation within the same subjects. There was no placebo arm, no control group, no randomization, and no blinding of participants or outcome assessors. A pre-post design of this kind cannot rule out regression to the mean, seasonal or temporal confounding, or a practice/repeat-testing effect on the flow-mediated dilation measurement.

Overall judgment: High, consistent with Table 1's existing "RoB: High (no control arm)" note for this study. This is the weakest evidentiary source among the probiotic clinical studies cited and should be described in the main text as hypothesis-generating pilot data, not as trial-level evidence.

Summary and Outstanding Actions

1. Resolved: El-Rakabawy et al. 2025 [ref. 40] carries a High risk-of-bias judgment (D2: no placebo/blinding). Section 5.3 of the main manuscript now states that, because this preliminary trial lacked a placebo control and blinding, its biomarker changes should be read as open-label findings rather than definitive evidence, consistent with this appraisal.

2. Resolved: Moludi et al. 2021 [ref. 29] full-text Methods confirm IRCT registration (IRCT20121028011288N15), blinded third-party allocation, and complete ITT accounting. Upgraded from "Some concerns" to "Low" overall — see updated write-up above.

3. Several ROBINS-I and SYRCLE judgments above rest on abstract-level or search-derived excerpts rather than full-text Methods sections (Zhang et al. 2025/26, Erlinge et al. 2025, Naso et al. 2025, Liu et al. 2025, Chen et al. 2025). Before submission, pull the full-text PDF of each and confirm or revise the “Unclear” domains accordingly — do not upgrade them to “Low” without checking the actual Methods text.

4. The simplified three-domain ROBINS-I adaptation used in Section B is a deviation from the full seven-domain instrument. If this table will appear in a formal risk-of-bias appendix for peer review, either apply the full ROBINS-I domain set or explicitly label the adaptation and cite a methodological justification for the simplification.

5. Across all three tools, this evidence base skews toward "Some concerns" / "Moderate" / "Unclear" rather than clean "Low" ratings. This is realistic and defensible for a mixed literature of five clinical trials (one confirmed Low via full-text verification, three "Some concerns" — two on abstract-level access, one on confirmed full-text — and one High for lack of blinding), one non-comparative pilot, four observational/imaging analyses, and five preclinical mouse studies — it should not be revised toward more favorable judgments without new supporting information from the primary sources.

6. Once each domain above has been checked against full-text sources, generate traffic-light figures (e.g., via the robvis R package) from these three tables for inclusion as supplementary figures, referenced explicitly from Section 2.3 of the main manuscript.

7. Resolved: Table 1 of the main manuscript now states "RoB: Some concerns" for both Jones et al. [ref. 32] and Szulińska et al. [ref. 35], matching this document's appraisal (Jones: D1/D3/D5; Szulińska: D1/D2/D3/D5, both based on abstract-level access). Table 1 and Table 2 also now carry full verdicts for Spasova et al. [ref. 27] and Bondonno et al. [ref. 42] ("Some concerns" and "Moderate," respectively, both grounded in confirmed full-text detail).

8. The SYRCLE judgments for Zhai et al. [ref. 34] and Qiu et al. [ref. 31] rest on abstract-level access, like Naso, Liu, and Chen above. Pull the full-text PDF of each before submission and confirm or revise the "Unclear" domains — do not upgrade them to "Low" without checking the actual Methods text.

9. Malik et al. [ref. 33] (Section D) is a single-arm pilot with no comparator and cannot be assessed by RoB 2, ROBINS-I, or SYRCLE. Its "High" judgment here is qualitative, not tool-derived. Ensure the main manuscript does not present its effect sizes (FMD improvement, IL-8/TNF-α reduction) alongside the RCT findings without flagging this design difference — Table 1 already does this correctly ("nonrandomized pilot" / "RoB: High, no control arm"), so no manuscript change is needed here, only confirmation that Section D's reasoning matches.

10. New since this document was last updated: several additional primary studies have been incorporated into the main manuscript that do not yet have entries here and have not been risk-of-bias appraised: Chung et al. 2016 [ref. 22] (UDCA/RAGE signaling, preclinical), Chong Nguyen et al. 2021 [ref. 23] (circulating bile acids and coronary artery disease, clinical/observational), Wang et al. 2012 [ref. 50] (cyanidin-3-O-β-glucoside, preclinical, ApoE−/− mice), Nishimoto et al. 2023 [ref. 54] (urolithin A, randomized controlled trial), and Istas et al. 2018 [ref. 55] (plasma urolithin metabolites, randomized controlled trial). These should be appraised using the same tools and transparency standard applied above (SYRCLE for the preclinical studies, ROBINS-I-adapted for the observational study, RoB 2 for the two RCTs) before this document is considered complete.
